# Supplementary material for: Monovalent mRNA XBB.1.5 vaccine effectiveness against COVID-19 hospitalization in Quebec, Canada: Impact of variant replacement and waning protection during 10-month follow-up
Source: PLoS One. 2025 Jun 3;20(6):e0325269. doi: 10.1371/journal.pone.0325269 (PMC12133164; doi:10.1371/journal.pone.0325269)
Supplement: S1 File — Contains Figure S1 (Flowchart), Figure S2 (Positive tests prior to vaccination), Table S1 (Characteristics of participants, XBB period), Table S2 (Characteristics of participants, JN period), Table S3 (Characteristics of participants, KP period), Table S4 (XBB-VE by time since vaccination), Table S5 (XBB-VE among participants with prior infection) and Table S6 (XBB-VE by age). (PDF) [file pone.0325269.s001.pdf]

Monovalent mRNA XBB.1.5 vaccine effectiveness against COVID-19 hospitalization in Quebec, Canada: impact of variant replacement and waning protection during 10-month follow-up

Sara Carazo, MD PhD – Danuta M. Skowronski, MD FRCPC – Nicholas Brousseau, MD MSc – Charles-Antoine Guay, MD M.Sc. FRCPC – Chantal Sauvageau, MD – Étienne Racine, MD – Denis Talbot, PhD – Iulia Gabriela Ionescu, MSc – Judith Fafard, MD – Rodica Gilca, MD PhD – Jonathan Phimmasone, MSc – Philippe De Wals, MD PhD – Gaston De Serres, MD PhD

Supplementary Material

S1 Figure. Flowchart of study population……………………………………….…………………3

S2 Figure. Temporal distribution of first and second positive SARS-CoV-2 nucleic acid amplification test prior to XBB-vaccination campaign, by participant vaccination status……………………………………………………………..…………………….….4

S1 Table. Characteristics of study participants during XBB period by outcome and vaccination status……………………………………..………………..………....5

S2 Table. Characteristics of study participants during JN period by outcome and vaccination status………………………………………………………………………..7

S3 Table. Characteristics of study participants during KP period by outcome and vaccination status………………………………………………………………...……9

S4 Table. XBB-vaccine effectiveness against COVID-19 hospitalization by time since XBB-vaccination and subvariant period………………………………………………..….11

S5 Table. XBB-vaccine effectiveness against COVID-19 hospitalization restricted to participants with prior NAAT-confirmed infection, by subvariant period…………………………………………………………..…………..………..….12

S6 Table. XBB-vaccine effectiveness against COVID-19 hospitalization, by age group and subvariant period……………….………………………………………………...….13

S1 Figure. Flowchart of study population

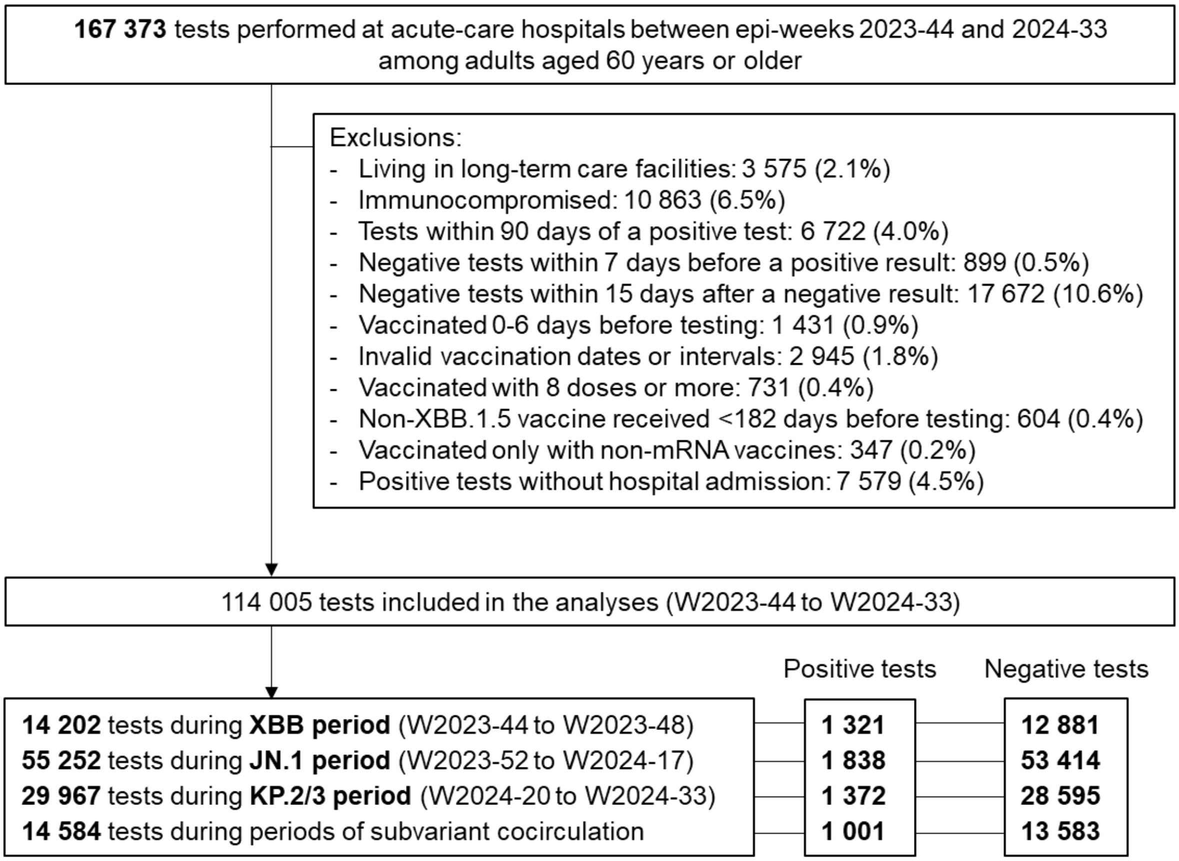

S2 Figure. Temporal distribution of first and second positive SARS-CoV-2 nucleic acid amplification test prior to XBB-vaccination campaign, by participant vaccination status

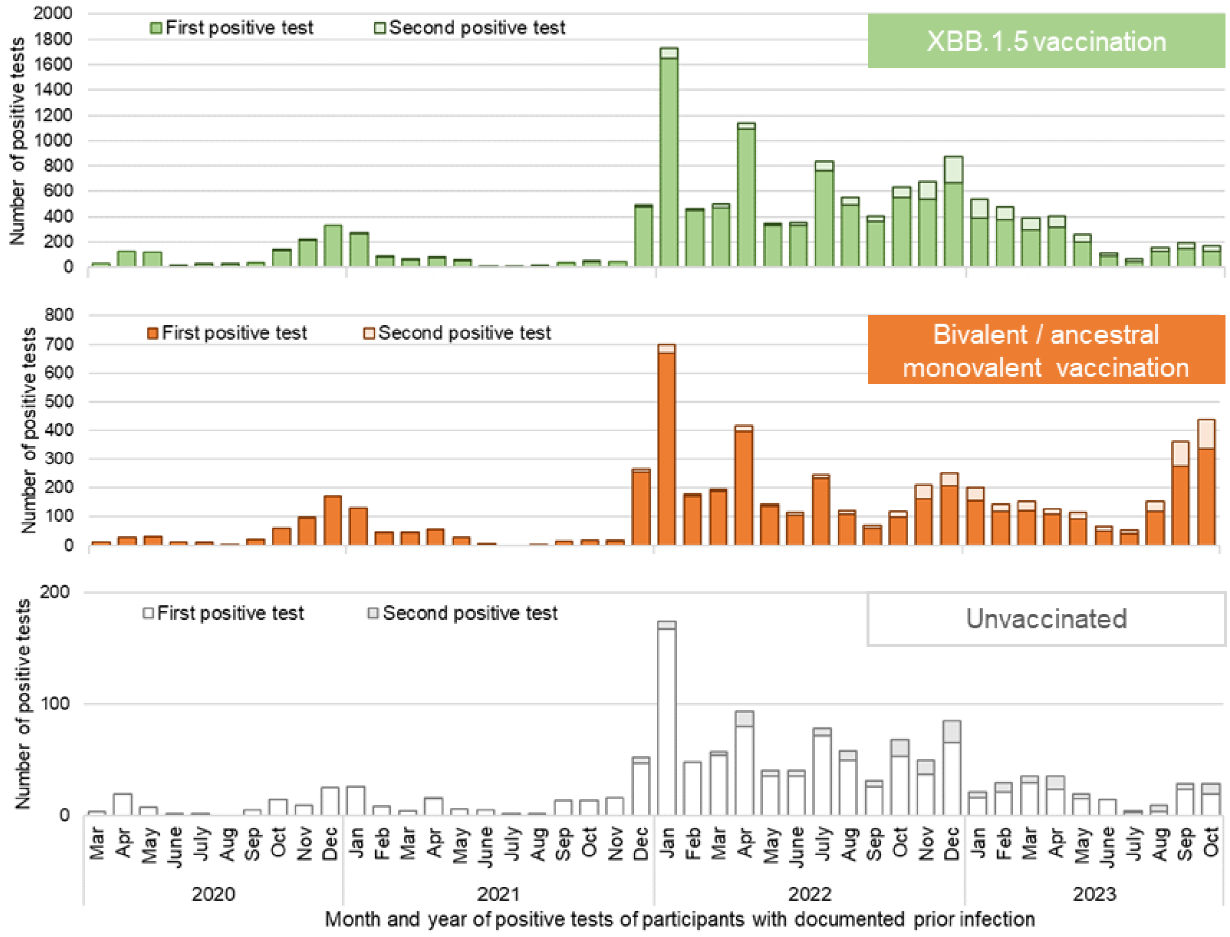

S1 Table. Characteristics of study participants during XBB period by outcome and vaccination status

XBB period	Cases		Controls	
**Characteristic**	**XBB vaccinated**	**MV/BV vaccinated**	**XBB vaccinated**	**MV/BV vaccinated**
**N**	201	556	3322	4384
**Sex**				
Female	89 (44.3)	277 (49.8)	1755 (52.8)	2285 (52.1)
Male	112 (55.7)	279 (50.2)	1567 (47.2)	2099 (47.9)
**Age, years**				
Mean (standard deviation)	82 (9.3)	81 (8.6)	79 (9.3)	78 (9.6)
60-69	21 (10.4)	55 (9.9)	664 (20.0)	1011 (23.1)
70-79	63 (31.3)	180 (32.4)	1129 (34.0)	1443 (32.9)
≥80	117 (58.2)	321 (57.7)	1529 (46.0)	1930 (44.0)
**Place of residence**				
Home	152 (75.6)	380 (68.3)	2455 (73.9)	3284 (74.9)
Private homes for older people	49 (24.4)	165 (29.7)	805 (24.2)	984 (22.4)
Other	0 (0.0)	11 (2.0)	62 (1.9)	116 (2.6)
**Chronic conditions**				
At least two chronic conditions[a]	174 (86.6)	432 (77.7)	2530 (76.2)	3235 (73.8)
Chronic heart disease	126 (62.7)	300 (54.0)	1608 (48.4)	2082 (47.5)
Chronic lung disease	80 (39.8)	188 (33.8)	1313 (39.5)	1575 (35.9)
Cancer	49 (24.4)	121 (21.8)	767 (23.1)	931 (21.2)
Neurologic disease / dementia	40 (19.9)	114 (20.5)	505 (15.2)	627 (14.3)
Obesity	22 (10.9)	54 (9.7)	412 (12.4)	519 (11.8)
**Documented prior infection history**	28 (13.9)	103 (18.5)	737 (22.2)	929 (21.2)
**Interval in months between prior infection and specimen collection,** median (IQR)	16.1 (13-20)	18.7 (12-22)	16.0 (11-21)	16.2 (10-22)
**Number of booster doses received**				
One (V3)	1 (0.5)	16 (2.9)	39 (1.2)	122 (2.8)
Two (V4)	10 (5.0)	172 (30.9)	199 (6.0)	1722 (39.3)
Three (V5)	44 (21.9)	365 (65.6)	767 (23.1)	2519 (57.5)
Four (V6)	113 (56.2)	3 (0.5)	1966 (59.2)	21 (0.5)
Five (V7)	33 (16.4)	0 (0.0)	351 (10.6)	0 (0.0)

**Interval in months between last dose and specimen collection,** median (IQR)	0.6 (0.4-1)	13.2 (12-14)	0.7 (0.5-1)	13.2 (12-14)
**2023/24 seasonal influenza vaccination** (≥14 days before specimen collection)	107 (53.2)	25 (4.5)	2120 (63.8)	245 (5.6)

Abbreviations: V3 to V7, total number of vaccine doses 3 to 7; MV/BV, monovalent or bivalent booster dose received between July and December 2022; IQR, interquartile range

[a] At least two chronic conditions among the following: chronic respiratory disease, hypertension, cardiovascular disease, neurological disorder, anaemia, diabetes, hypothyroidism, fluid and electrolyte disorders, cancer, kidney disease, obesity, psychosis, liver disease, coagulopathy, weight loss, drug abuse, alcohol abuse, ulcer, paralysis.

Note: Numbers are n and column proportions except otherwise indicated

S2 Table. Characteristics of study participants during JN period by outcome and vaccination status

JN period	Cases		Controls	
**Characteristic**	**XBB vaccinated**	**MV/BV vaccinated**	**XBB vaccinated**	**MV/BV vaccinated**
**N**	814	363	27041	9069
**Sex**				
Female	387 (47.5)	176 (48.5)	14141 (52.3)	4568 (50.4)
Male	427 (52.5)	187 (51.5)	12900 (47.7)	4501 (49.6)
**Age, years**				
Mean (standard deviation)	80 (9.0)	79 (8.9)	79 (9.3)	77 (9.6)
60-69	111 (13.6)	59 (16.3)	4986 (18.4)	2520 (27.8)
70-79	271 (33.3)	129 (35.5)	9057 (33.5)	3040 (33.5)
≥80	432 (53.1)	175 (48.2)	12998 (48.1)	3509 (38.7)
**Place of residence**				
Home	597 (73.3)	296 (81.5)	19523 (72.2)	7348 (81.0)
Private homes for older people	198 (24.3)	62 (17.1)	6836 (25.3)	1525 (16.8)
Other	19 (2.3)	5 (1.4)	682 (2.5)	196 (2.2)
**Chronic conditions**				
At least two chronic conditions[a]	631 (77.5)	272 (74.9)	20466 (75.7)	6418 (70.8)
Chronic heart disease	408 (50.1)	161 (44.4)	13045 (48.2)	3910 (43.1)
Chronic lung disease	309 (38.0)	128 (35.3)	10538 (39.0)	3284 (36.2)
Cancer	190 (23.3)	69 (19.0)	5895 (21.8)	1802 (19.9)
Neurologic disease / dementia	164 (20.1)	62 (17.1)	4014 (14.8)	1108 (12.2)
Obesity	85 (10.4)	45 (12.4)	3157 (11.7)	1034 (11.4)
**Documented prior infection history**	145 (17.8)	58 (16.0)	5946 (22.0)	2262 (24.9)
**Interval in months between prior infection and specimen collection,** median (interquartile range)	18.5 (14-23)	17.6 (6-24)	18.9 (14-24)	13.8 (5-24)
**Number of booster doses received**				
One (V3)	14 (1.7)	16 (4.4)	309 (1.1)	309 (3.4)
Two (V4)	48 (5.9)	151 (41.6)	1822 (6.7)	4362 (48.1)
Three (V5)	168 (20.6)	195 (53.7)	6831 (25.3)	4367 (48.2)
Four (V6)	472 (58.0)	1 (0.3)	15028 (55.6)	31 (0.3)
Five (V7)	112 (13.8)	0 (0.0)	3051 (11.3)	0 (0.0)

**Interval in months between last dose and specimen collection,** median (interquartile range)	2.5 (2-4)	15.4 (14-17)	3.3 (2-5)	16.3 (15-18)
**2023/24 seasonal influenza vaccination** (≥14 days before specimen collection)	651 (80.0)	65 (17.9)	21431 (79.3)	2164 (23.9)

Abbreviations: V3 to V7, total number of vaccine doses 3 to 7; MV/BV, monovalent or bivalent booster dose received between July and December 2022; IQR, interquartile range

[a] At least two chronic conditions among the following: chronic respiratory disease, hypertension, cardiovascular disease, neurological disorder, anaemia, diabetes, hypothyroidism, fluid and electrolyte disorders, cancer, kidney disease, obesity, psychosis, liver disease, coagulopathy, weight loss, drug abuse, alcohol abuse, ulcer, paralysis.

Note: Numbers are n and column proportions except otherwise indicated

S3 Table. Characteristics of study participants during KP period by outcome and vaccination status

KP period	Cases		Controls	
**Characteristic**	**XBB vaccinated**	**MV/BV vaccinated**	**XBB vaccinated**	**MV/BV vaccinated**
**N**	781	204	15401	4299
**Sex**				
Female	388 (49.7)	106 (52.0)	7949 (51.6)	2142 (49.8)
Male	393 (50.3)	98 (48.0)	7452 (48.4)	2157 (50.2)
**Age, years**				
Mean (standard deviation)	81 (8.8)	80 (8.7)	79 (9.2)	76 (9.3)
60-69	90 (11.5)	26 (12.7)	2776 (18.0)	1239 (28.8)
70-79	221 (28.3)	70 (34.3)	5361 (34.8)	1530 (35.6)
≥80	470 (60.2)	108 (52.9)	7264 (47.2)	1530 (35.6)
**Place of residence**				
Home	581 (74.4)	174 (85.3)	11577 (75.2)	3680 (85.6)
Private homes for older people	188 (24.1)	26 (12.7)	3466 (22.5)	520 (12.1)
Other	12 (1.5)	4 (2.0)	358 (2.3)	99 (2.3)
**Chronic conditions**				
At least two chronic conditions[a]	621 (79.5)	148 (72.5)	11502 (74.7)	2925 (68.0)
Chronic heart disease	399 (51.1)	95 (46.6)	7230 (46.9)	1775 (41.3)
Chronic lung disease	293 (37.5)	61 (29.9)	5890 (38.2)	1515 (35.2)
Cancer	169 (21.6)	38 (18.6)	3457 (22.4)	866 (20.1)
Neurologic disease / dementia	130 (16.6)	24 (11.8)	2128 (13.8)	449 (10.4)
Obesity	99 (12.7)	21 (10.3	1698 (11.0)	489 (11.4)
**Documented prior infection history**	158 (20.2)	28 (13.7)	3614 (23.5)	1034 (24.1)
**Interval in months between prior infection and specimen collection,** median (interquartile range)	23.6 (19-29)	25.3 (10-28)	21.2 (15-28)	15.8 (8-28)
**Number of booster doses received**				
One (V3)	2 (0.3)	9 (4.4)	183 (1.2)	166 (3.9)
Two (V4)	54 (6.9)	91 (44.6)	987 (6.4)	2209 (51.4)
Three (V5)	174 (22.3)	104 (51.0)	3620 (23.5)	1913 (44.5)
Four (V6)	406 (52.0)	0 (0.0)	8152 (52.9)	11 (0.3)
Five (V7)	145 (18.6)	0 (0.0)	2459 (16.0)	0 (0.0)

**Interval in months between last dose and specimen collection,** median (interquartile range)	8.0 (7-9)	20.9 (20-22)	7.2 (6-8)	20.6 (20-22)
**2023/24 seasonal influenza vaccination** (≥14 days before specimen collection)	634 (81.2)	52 (25.5)	12004 (77.9)	1016 (23.6)

Abbreviations: V3 to V7, total number of vaccine doses 3 to 7; MV/BV, monovalent or bivalent booster dose received between July and December 2022; IQR, interquartile range

[a] At least two chronic conditions among the following: chronic respiratory disease, hypertension, cardiovascular disease, neurological disorder, anaemia, diabetes, hypothyroidism, fluid and electrolyte disorders, cancer, kidney disease, obesity, psychosis, liver disease, coagulopathy, weight loss, drug abuse, alcohol abuse, ulcer, paralysis.

Note: Numbers are n and column proportions except otherwise indicated

S4 Table. XBB-vaccine effectiveness against COVID-19 hospitalization by time since XBB vaccination and subvariant period

	XBB period	JN period	KP period
	Adjusted VE[a] (%) (95% CI)	Adjusted VE[a] (%) (95% CI)	Adjusted VE[a] (%) (95% CI)
Global	54.4 (45.9 to 61.6)	22.7 (12.1 to 32.0)	0.5 (-16.7 to 15.2)
**Time since last XBB-vaccination**			
7-30d (month 1)	53.2 (43.5 to 61.3)	27.8 (3.4 to 46.0)	67.4 (34.7 to 83.7)
31-60d (month 2)	60.4 (46.0 to 70.9)	22.5 (6.2 to 36.0)	57.2 (29.3 to 74.1)
61-91d (month 3)	NE	27.6 (14.5 to 38.8)	31.0 (-6.0 to 55.0)
92-121d (month 4)	NE	19.8 (0.2 to 35.5)	35.7 (-12.9 to 63.4)
122-152d (month 5)	NE	23.1 (-3.1 to 42.6)	44.7 (-19.6 to 74.5)
153-182d (month 6)	NE	6.5 (-44.1 to 39.3)	3.4 (-40.4 to 33.5)
183-213d (month 7)	NE	-5.2 (-159.5 to 57.4)	11.9 (-16.3 to 33.2)
214-243d (month 8)	NE	NE	-2.1 (-26.8 to 17.9)
244-274d (month 9)	NE	NE	-19.4 (-47.3 to 3.2)
275-304d (month 10)	NE	NE	-14.2 (-49.2 to 12.7)

Abbreviations: CI, confidence interval; d, days; NE, not estimable; VE, vaccine effectiveness.

[a] Logistic regression model comparing XBB-vaccinated with participants last vaccinated with ancestral monovalent or bivalent vaccines from July to December 2022 and adjusted for sex, age group, chronic conditions, place of residence and epi-week (2-week periods)

S5 Table. XBB-vaccine effectiveness against COVID-19 hospitalization restricted to participants with prior NAAT-confirmed infection, by subvariant period

Subvariant period and comparison groups	Cases	Controls	Unadjusted VE (%) (95% CI)	Adjusted VE[a] (%) (95% CI)
**Global (W2023-44 to W2024-33)**				
XBB-vaccinated and prior infection	378	11033		
vs MV/BV-vaccinated in 2022 regardless prior infection status	1356	20346	48.6 (42.3 to 54.2)	43.4 (35.9 to 50.1)
vs MV/BV-vaccinated in 2022 with documented prior infection	216	3906	38.0 (26.5 to 47.8)	28.2 (13.6 to 40.3)
**XBB period**				
XBB-vaccinated and prior infection	29	752		
vs MV/BV-vaccinated in 2022 regardless prior infection status	556	4386	69.6 (55.5 to 79.2)	73.6 (61.1 to 82.1)
vs MV/BV-vaccinated in 2022 with documented prior infection	103	931	65.1 (46.8 to 77.2)	65.5 (46.8 to 77.6)
**JN period**				
XBB-vaccinated and prior infection	146	5811		
vs MV/BV-vaccinated in 2022 regardless prior infection status	363	9070	37.2 (23.7 to 48.3)	41.1 (27.5 to 52.1)
vs MV/BV-vaccinated in 2022 with documented prior infection	53	1747	17.2 (-13.9 to 39.8)	14.1 (-18.8 to 37.9)
**KP period**				
XBB-vaccinated and prior infection	145	3104		
vs MV/BV-vaccinated in 2022 regardless prior infection status	204	4299	1.6 (-22.4 to 20.8)	5.9 (-19.3 to 25.8)
vs MV/BV-vaccinated in 2022 with documented prior infection	23	693	-10.8 (-120.2 to 10.0)	-24.9 (-97.3 to 21.0)

Abbreviations: CI, confidence interval; MV/BV, ancestral monovalent or bivalent booster dose received between July and December 2022; NAAT, nucleic acid amplification test; VE, vaccine effectiveness; W, week

[a] Logistic regression model comparing XBB-vaccinated with participants last vaccinated with ancestral monovalent or bivalent vaccines from July to December 2022 and adjusted for sex, age group, chronic conditions, place of residence and epi-week

S6 Table. XBB-vaccine effectiveness against COVID-19 hospitalization, by age group and subvariant period

	Age group		
	**60-69-year-olds**	**70-79-year-olds**	**≥80-year-olds**
	Adjusted VE[a] (%) (95% CI)	Adjusted VE[a] (%) (95% CI)	Adjusted VE[a] (%) (95% CI)
**Global**	3.9 (-17.7 to 21.5)	33.4 (24.1 to 41.5)	33.5 (26.2 to 40.0)
**XBB period**			
Any time since XBB-vaccination	47.5 (10.5 to 69.2)	56.1 (40.6 to 67.6)	54.9 (43.3 to 64.1)
7-60d since XBB-vaccination	47.5 (10.5 to 69.2)	56.1 (40.6 to 67.6)	54.9 (43.3 to 64.1)
**JN period**			
Any time since XBB-vaccination	-6.7 (-47.5 to 22.8)	25.5 (7.5 to 40.1)	28.8 (14.5 to 40.7)
7-60d since XBB-vaccination	-23.7 (-91.7 to 20.2)	34.7 (11.1 to 52.0)	27.2 (6.2 to 43.6)
**KP period**			
Any time since XBB-vaccination	-47.2 (-130.3 to 5.9)	10.3 (-18.5 to 32.0)	6.6 (-16.3 to 25.0)
7-60d since XBB-vaccination	53.2 (-136.2 to 90.7)	55.9 (-7.8 to 82.0)	63.0 (28.3 to 77.9)

Abbreviations: CI, confidence interval; d, days; VE, vaccine effectiveness.

[a] Logistic regression model comparing XBB-vaccinated participants by number of XBB doses (global or at two-month intervals from vaccination) with participants last vaccinated with ancestral monovalent or bivalent vaccines from July to December 2022, and adjusted for sex, chronic conditions, place of residence and epi-week (2-week periods)
